# A new therapy (MP29-02*) provides effective relief from all individual nasal and ocular symptoms of seasonal allergic rhinitis

**DOI:** 10.1186/2045-7022-3-S2-P41

**Published:** 2013-07-16

**Authors:** Peter Hellings, Claus Bachert, Jean Bousquet, Glenis Scadding, Wytske Fokkens, Ullrich Munzel, David Price

**Affiliations:** 1 University Hospitals Leuven, Leuven, Belgium; 2 Ghent University Hospital, Dept of Oto-rhinolaryngology, Ghent, Belgium; 3 Hopital Arnaud de Villeneuve University Hospital, Montpellier, France; 4 The Royal National Throat, Nose and Ear Hospital, London, UK; 5 Academic Medical Center, Dept of Otorhinolaryngology, Amsterdam, the Netherlands; 6 Meda Pharma, Biostatistics & Market Access, Bad Homburg, Germany; 7 Univserity of Aberdeen, Dept of General Practice & Primary Care, Aberdeen, UK

## Background

Congestion is reported by allergic rhinitis (AR) patients as the most bothersome nasal symptom. However, ocular symptoms have the greatest impact on patient quality of life. A novel treatment which more effectively controls both nasal and ocular symptoms would fill the gap for an important unmet need in AR.

## Objective

To assess the efficacy of MP29-02* (a novel intranasal formulation of azelastine hydrochloride [AZE] and fluticasone propionate [FP]) in providing relief from each of the nasal and ocular symptoms commonly experienced by patients with seasonal AR (SAR) compared to commercially available intranasal AZE or FP nasal sprays or placebo.

## Methods

Patients (≥12 years old) with moderate-to-severe SAR (n=610) were randomized into this double-blind, placebo-controlled, 14-day, parallel-group trial to MP29-02*, commercially-available AZE or FP nasal sprays, or placebo (all given as 1 spray/nostril bid [total daily dose: AZE 548µg; FP 200µg]). The primary efficacy variable was change from baseline in reflective total nasal symptom score (rTNSS; AM +PM) over 14-days. Secondary endpoints included change from baseline in each of the individual nasal and ocular symptoms.

## Results

MP29-02* reduced nasal congestion by -1.24 vs -0.86 for FP (Diff: -0.39; 95% CI -0.65, -0.13; p=0.0034), -0.75 for AZE (Diff: -0.49; 95% CI: -0.74, -0.24; p=0.0001) and -0.54 for placebo (Diff: -0.70; 95% CI -0.95, -0.45; p<0.0001), with a relative difference of 54% to FP and 70% to AZE. A similar and significant superiority of MP29-02* over FP and AZE was observed for nasal itching (44% to FP, 56% to AZE), rhinorrhoea (32% to FP; 65% to AZE) and sneezing (49% to FP; 61% to AZE). The most bothersome ocular symptom of itching was reduced by -1.23 by MP29-02* vs -0.70 for FP (Diff: -0.53; 95% CI: -0.79, -0.26; p=0.0001), -0.88 for AZE (Diff: -0.35; 95% CI -0.63, -0.08; p=0.0127) and -0.44 for placebo (Diff: -0.79, 95% CI -1.04, -0.54; p<0.0001) with a relative difference of 67% to FP and 44% to AZE. The relative differences to FP and AZE respectively were 51% and 25% for ocular watering, and 53% and 40% for ocular redness.

## Conclusion

MP29-02* provides superior relief from each nasal and ocular symptom compared to first line AR therapies, including the most bothersome symptoms of nasal congestion and ocular itching, and can be considered the drug of choice for AR.

*Dymista

